# Space–Time Patterns of Poultry Pathogens in the USA: A Case Study of *Ornithobacterium rhinotracheale* and *Pasteurella multocida* in Turkey Populations

**DOI:** 10.3390/pathogens12081004

**Published:** 2023-07-31

**Authors:** Magnus R. Campler, Amro Hashish, Mostafa Ghanem, Mohamed M. El-Gazzar, Andréia G. Arruda

**Affiliations:** 1 Department of Veterinary Preventive Medicine, College of Veterinary Medicine, The Ohio State University, Columbus, OH 43210, USA; campler.1@osu.edu; 2 Department of Veterinary Diagnostic and Production Animal Medicine, College of Veterinary Medicine, Iowa State University, Ames, IA 50011, USA; hashish@iastate.edu (A.H.); elgazzar@iastate.edu (M.M.E.-G.); 3 Department of Veterinary Medicine, College of Veterinary Medicine, University of Maryland, College Park, MD 20740, USA; mghanem@umd.edu

**Keywords:** infectious disease, spatio-temporal clusters, mapping, poultry

## Abstract

Respiratory infections caused by *Ornithobacterium rhinotrachealis* (ORT) and *Pasteurella multocida* (PM) bacteria are significant threats to the poultry industry by causing economic losses and welfare issues. Due to characterization difficulties and underutilization of epidemiological tools, description of the spatio-temporal spread of these diseases in the field is limited. The objectives of this retrospective observational cross-sectional study were to (a) investigate the existence of space–time clusters (hotspots); and (b) investigate the association between genetic similarity and spatial proximity for both pathogens using molecular typing and a recently developed Core-Genome Multilocus Sequencing Typing (cgMLST) scheme. ORT (*n* = 103) and PM (*n* = 69) isolates from confirmed disease outbreaks from one commercial company between 2013 and 2021 were obtained from a veterinary diagnostic laboratory, characterized using a cgMLST scheme and visualized using a minimum spanning tree. Spatio-temporal cluster analysis using SaTScan^TM^ and a Spearman’s rank correlation were performed to investigate clustering and any association between allelic diversity and geospatial distance. The cgMLST sequencing revealed three allelic clusters for ORT and thirteen clusters for PM. The spatio-temporal analysis revealed two significant clusters for PM, one with a 259.3 km cluster containing six cases between May and July 2018 and a 9 km cluster containing five cases between February 2019 and February 2021. No spatio-temporal clusters were found for ORT. A weak negative correlation between allelic diversity and geospatial distance was observed for ORT (r = −0.04, *p* < 0.01) and a weak positive correlation was observed for PM (r = 0.11, *p* < 0.01). This study revealed regional spatio-temporal clusters for PM in commercial turkey sites between 2018 and 2021 and provided additional insight into bacterial strain subgroups and the geographical spread of ORT and PM over time.

## 1. Introduction

Respiratory infections caused by *Ornithobacterium rhinotrachealis* (ORT) and *Pasteurella multocida* (PM) bacteria are significant threats to the turkey industry by causing economic losses and associated welfare issues [1,2]. Currently, both ORT [3] and PM are ranked among the most problematic global re-emerging turkey diseases [4]. Exposure and infection with ORT can lead to increased mortality, growth suppression and decreased egg production [5], and PM can cause septicemia and subsequent death [6]. In general, both ORT [7] and PM [4] are often associated with secondary infections. However, PM is often considered a primary pathogen [8], while the synergistic tendencies of ORT make it likely to act as a secondary opportunistic pathogen [9,10]. ORT is often isolated during respiratory disease caused by other pathogens such as avian pneumovirus [11], avian influenza H9N2 [12] and Newcastle disease [13], and a recent study by [14] suggested a link between gut microbiota and the emergence of ORT in turkeys with impaired weight gain. To date, ORT [15] and PM [16,17] remain a challenge in terms of timely identification, treatment and the prevention. In addition, PM has been more thoroughly investigated compared to ORT which is reflected by the seven complete avian ORT genomes available in GenBank^®^ (https://www.ncbi.nlm.nih.gov/genome/browse/#!/prokaryotes/10734/, accessed on 7 July 2023) [18] compared to the 139 complete genomes for PM (https://www.ncbi.nlm.nih.gov/genome/browse/#!/prokaryotes/912/, accessed on 27 July 2023). The use of whole genome sequencing enables further isolate identification and serological characterization of genotypes including steps such as multi-loci sequence typing, virulence factor-encoding genes and whole genome phylogeny to establish evolutionary relationships and clonality among commercial turkey populations [2,19,20,21]. Having a robust way to capture and analyze data upon the detection of emerging pathogens is of outmost importance to allow for a timely mitigation response for the prevention of disease spread. Recently, a TaqMan Real-Time Polymerase Chain Reaction (qPCR) has been developed for ORT [22] and a unique Core-Genome Multi-Locus Sequencing Typing cgMLST technique, tailored specifically for ORT [23] and PM [24] has recently been developed to aid in the characterization of these pathogens.

There are several aspects that make ORT and PM attractive models for epidemiological method applications: on one hand, ORT is relatively recently recognized as an emerging threat to the poultry industry [7,25] and most of its epidemiology (e.g., transmission routes, pathogen role) is still unclear. On the other hand, PM is a well-recognized disease within the poultry industry, with ample literature describing its pathogeneses and transmission routes [21,26,27]. Both PM and ORT affect multiple species, which makes them a greater concern within a larger demographic among different animal populations [5,28]. A thorough understanding of disease epidemiology, including spatial and temporal trends is an essential component in establishing the frameworks for the prevention, control and eradication of future poultry disease outbreaks. Spatio-temporal analysis allows the visualization of disease spread geographically over time. Previous research investigated the effect of environmental factors on PM within the USA [29,30,31]; however, the combination of molecular characterization with spatio-temporal analyses for PM and ORT in poultry is currently lacking in the peer-reviewed literature.

Thus, the objectives of this study were as follows: (1) to investigate the space–time patterns in the region through cluster analyses, and (2) to investigate the association between genetic similarity and spatial proximity for ORT and PM in the midwestern United States using information from ORT and PM isolates collected from commercial turkey sites with respiratory disease typed via a recently developed cgMLST scheme.

## 2. Materials and Methods

### 2.1. Study Period, Study Area and Sample Collection

This study utilized a retrospective observational cross-sectional study design based on the ORT (*n* = 103) and PM (*n* = 69) clinical isolates obtained from commercial turkey sites in the midwestern United States between 2013 and 2021. All sites belonged to the same production company and coordinates (latitude/longitude) were obtained for each site. An institutional animal care and use committee (IACUC) review and approval was not deemed necessary as all obtained and analyzed data were part of routine health management for disease screening and to confirm suspected outbreaks of ORT or PM under a veterinarian–client relationship; and the study was all retrospectively conducted. Sampling and isolate management was conducted under the guidance of the residing flock veterinarians at the production company and confirmed positive isolates were kept frozen in an isolate storage bank at the Animal Disease Diagnostic Laboratory (ADDL) at the Ohio Department of Agriculture (ODA) for later analysis. Only one confirmed isolate was available from the year 2013 for both ORT and PM, and no isolates were available for 2015 and 2017 for ORT.

### 2.2. Molecular Characterization

For high-resolution molecular characterization of ORT and PM, two recently developed cgMLST schemes [23,24] were utilized. The two cgMLST schemes were developed using Ridom SeqSphere + software version 8.3.1 (Munster, Germany, http://www.ridom.de/seqsphere accessed on 27 July 2023) [21]. Briefly, for ORT, the genome of strain ORT-UMN88 (CP006828.1) was used as a reference strain. Additionally, 32 ORT-diverse genomes were selected to develop the scheme. Eleven out of the 32 genomes were downloaded from GenBank and 21 were sequenced in this study (Supplementary Table S3). As described in a previous study, bacterial culture was performed on blood agar (with 5% sheep blood), and the cultures were incubated under microaerophilic conditions at 37 °C for 48 h. Isolates were identified and confirmed as ORT using real-time PCR [22]. Extraction of bacterial DNA was performed using 100 µL of the bacterial isolates’ suspension using a MagMAX™ Pathogen RNA/DNA Kit (Thermo Fisher Scientific, Waltham, MA, USA) on a Kingfisher-Flex instrument (Thermo Fisher Scientific) following the instructions of the manufacturer. Nucleic acids were eluted into 90 µL of elution buffer. DNA extracts from isolates were quantified using a Qubit fluorometric analysis double-stranded DNA HS (high sensitivity) scheme kit (Invitrogen, Waltham, MA, USA). Subsequently, ORT isolates were submitted to The Molecular Epidemiology Laboratory at the University of Maryland for WGS using an Illumina MiSeq sequencer (Illumina, San Diego, CA, USA) and the genome libraries were constructed using the Nextera XT DNA sample prep kit (Illumina). Generated WGS were assembled using SPAdes Genome Assembler implemented within BV-BRC [32]. All the generated genome assemblies were evaluated using QUAST [33]. The cgMLST scheme was composed using the cgMLST target definer tool, using the default settings within the Ridom SeqSphere + software. FASTA files of ORT genome assemblies were loaded into the software. Only contigs of the genomes > 200 bp were included in the analysis. A filtration step of unfit gene targets for cgMLST was applied; this filtration step included: a homologous gene filter to exclude all genes with high DNA similarity within a genome (with 90% identity and 100 bp overlap); a start codon filter to exclude all genes that were devoid of the translation start codon at the beginning of the gene; a minimum-length filter to exclude all genes with length of 50 bp; a stop codon filter to exclude all genes that were devoid of stop codons, had multiple stop codons, or had a stop codon that was not located at the end of the gene; and a gene overlap filter that excluded the shorter of two genes overlapping by >4 [34,35,36,37]. A total of 1250 genes were defined as core genome targets, representing 54.3% of the genome sequence.

For PM, the genome of strain Pm70 (NC_002663.1) was used as the reference strain. Moreover, a total of 96 PM genomes (64 were downloaded from GenBank and 32 were sequenced in this study, Supplementary Table S3) covering wide geographical locations (countries from 5 continents) across a broad time span (1962–2021) and from different avian species were selected to develop the PM-cgMLST. Bacterial cultures of PM isolates were conducted by streaking the original isolates on blood medium plates and incubating at 37 °C [38]. Characterization of the obtained isolates was performed by matrix-assisted laser desorption ionization time of flight mass spectrometry (MALDI-TOF MS) [39]. Afterwards, PM isolates were submitted to The Molecular Epidemiology Laboratory at the University of Maryland for WGS following the same methods as previously described for the ORT isolates. The number of core genome targets for PM was 1259 genes, representing 62.1% of the genome sequence. To enhance the ability of developed cgMLST schemes to differentiate between epidemiologically related and unrelated strains, a cluster typing threshold—the maximum number of allele differences that can be found between highly clonal outbreak samples—was 21 for the ORT and 120 for the PM. A minimum spanning tree analysis was created from the characterized isolate allelic differences and the allelic cut-off (>50 for the ORT and >120 for the PM) was used to distinguish genomic clusters.

All isolate meta-data was extracted and consolidated in Excel^®^ (Microsoft Corporation, Redmond, WA, USA) and later imported to a open-source geographic information system software QGIS 3.22.16 (QGIS Geographic Information System. QGIS Association. http://www.qgis.org accessed on 27 July 2023) for map plotting and visualization.

### 2.3. Statistical Analysis

A spatio-temporal cluster analysis was performed using SaTScan™ software, version 10.1 (SaTScan^TM^, Boston, MA, USA, http://www.satscan.org accessed on 27 July 2023), using a Kulldorff retrospective space–time permutation probability model with a circular scanning window across space and time for high incidence rates. Cases were analyzed on a monthly level to identify ORT and PM clusters using confirmed positive cases across between 2013 and 2021 [40]. The number of observed cases within a cluster were compared to the distribution of expected cases with the null hypothesis being based on that spatial and temporal location of all cases being independent. All models were fitted and evaluated through Monte Carlo simulations (999 replications). The site coordinates were used as spatial information and the maximum size of a spatial cluster was determined as 50% of the population at risk and 50% of the study period. *p*-values < 0.05 were considered statistically significant.

Finally, a Spearman’s rank correlation was conducted to investigate any association between the core gene allelic differences between sub-clusters of ORT and PM and their geospatial distance. *p*-values < 0.05 were considered statistically significant.

## 3. Results

### 3.1. Allelic Clusters and Diversity

**Figure 1:**
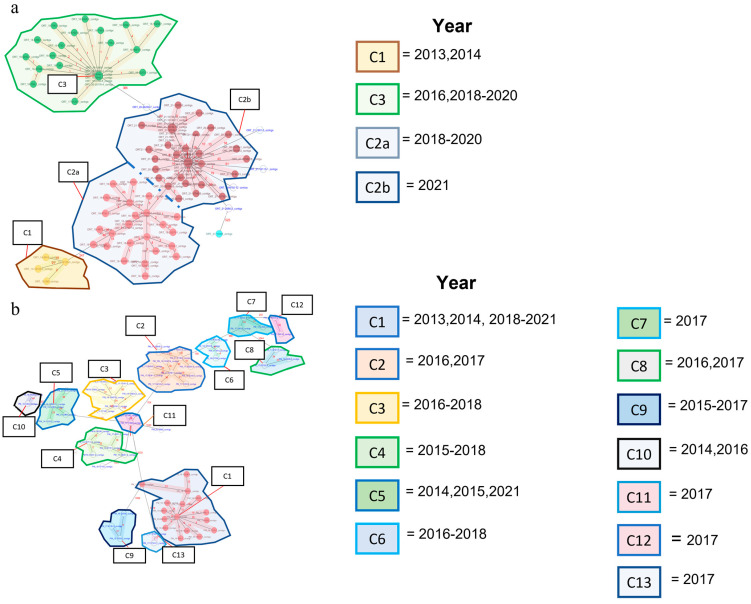
Minimum spanning tree analysis of *Ornithobacterium rhinotrachealis* (ORT) (**a**) and *Pasteurella multocida* (PM) (**b**) isolates based on the allelic profiles of 1250 and 1259 single-copy core genome targets, respectively. Clusters are abbreviated as C followed by cluster number. Each cluster represents isolates with a closely related allelic profile from other clusters using an allelic cut-off value for the genetic association as: ORT > 50 and PM >120.

The minimum spanning tree analysis revealed three allelic main clusters for ORT (Cluster 1, *n* = 4; Cluster 2, *n* = 70, and Cluster 3, *n* = 23; Figure 1a). Six isolates were classified as singletons (i.e., could not be classified within any of the existing three clusters). Cluster 2 contained subgroupings of isolates below the preset allelic threshold and was therefore divided into two distinct subclusters (C2a, *n* = 28, and C2b, *n* = 42; Figure 1a). For PM, the minimum spanning tree analysis revealed 13 distinct clusters (Figure 1b).

The number of isolates (Ni) and the number of isolate comparisons (Nc) as well as the median, min and max allelic differences for ORT are provided in Supplementary Table S1. Between 2013 and 2017, only seven confirmed ORT outbreaks were observed across all sites. An increase in outbreaks was reported between 2018 and 2021 likely due to internal contamination between the sites within the production system. Similarly, an increase in the median allelic diversity was observed in 2018 and was likely due to an overlapping genetic clustering and influx of distantly related isolates among sites. This change was represented in the increase in the maximum allelic differences within each year from 2018 and onwards.

**Figure 2:**
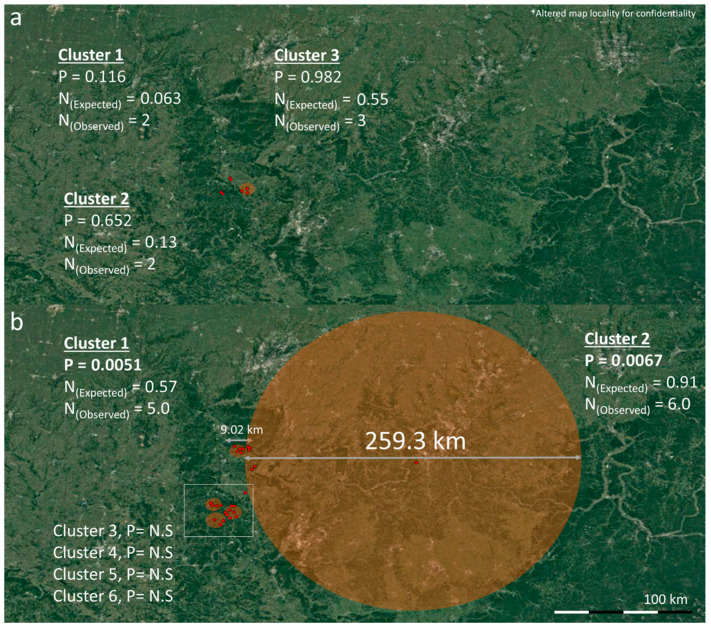
Significant spatio-temporal clusters (*n* = 0) for *Ornithobacterium rhinotrachealis* (ORT) (**a**) and *Pasteurella multocida* (PM) (*n* = 2) (**b**) between 2013 and 2021 using SaTScan 10.1. Significance was considered *p* < 0.05.

**Figure 3:**
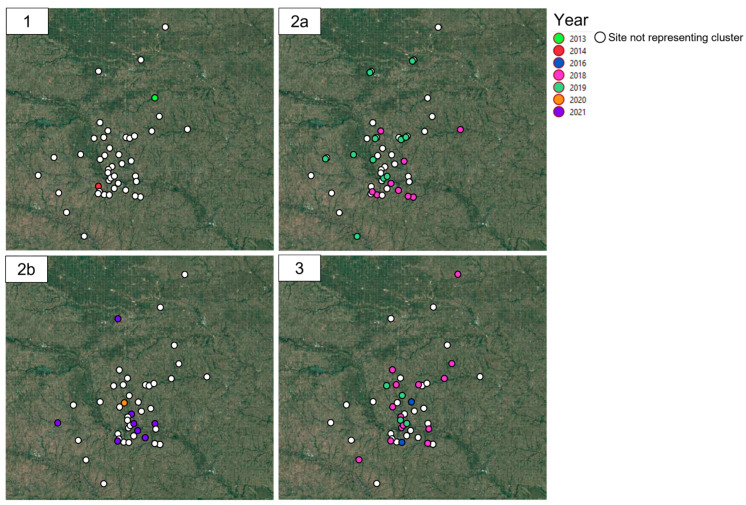
Spatial visualization of confirmed outbreaks of *Ornithobacterium rhinotrachealis* by year (2013–2021) and cluster (**1**, **2a**, **2b**, and **3**) identified based on the allelic profile of a 1250 single-copy core genome target and an allelic cut-off value of >50 using the open-source geographic information system software QGIS 3.22.16. Each panel represents one genetic cluster type.

The number of Isolates (Ni) and the number of isolate comparisons (Nc) as well as the median, min and max allelic differences for PM are provided in Supplementary Table S2. Across the years, the reported PM isolates represented a high degree of genetic diversity among the clusters except for the isolates reported in 2019, for which the multiple reported outbreaks belonged to the same or closely related isolates within cluster 1 (Figure 1 and Figure 2b; Figure 3—panel 1).

### 3.2. Spatio-Temporal Analysis

**Figure 4:**
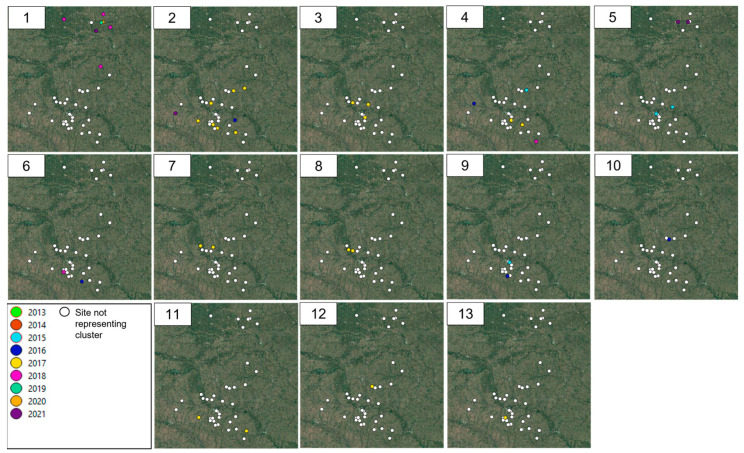
Spatial visualization of confirmed outbreaks of *Pasteurella multocida* by year (2013–2021) and cluster (**1**–**13**) identified based on the allelic profile of a 1259 single-copy core genome target and an allelic cut-off value of >120 using the open-source geographic information system software QGIS 3.22.16. Each panel represents one genetic cluster type.

No spatio-temporal clusters were detected for ORT (*p* > 0.05) (Figure 2a). For PM, two spatio-temporal clusters were detected. One cluster was detected between May and July 2018 with six observed cases within a 259.3 km radius (test statistic = 6.508) and a second spatio-temporal cluster with five cases from February 2019 to February 2021 within a 9 km radius (test statistic = 6.652) (Figure 2b). The geographical location for each confirmed outbreak and year within clusters in the study area are visualized in Figure 3 and Figure 4 for ORT and PM, respectively. The outbreak data is superimposed on an altered geographic map while maintaining the cluster data relative distance and accuracy to protect the site’s confidentiality.

The Spearman’s rank correlation showed a weak negative correlation between the allelic diversity and geospatial distance for ORT (r = −0.04, *p* = 0.01). This is indicative of continuous in-between site transfers for ORT spread and reduced genomic diversity with increased distance from the initial outbreak location for this commercial poultry system. In contrast, a weak positive correlation was observed for PM (r = 0.11, *p* < 0.01) indicative of limited in-between site transfers throughout the production company with increased genomic plasticity with increased geographical distance from the initial outbreak location.

## 4. Discussion

Our study explored the epidemiology of two important pathogens in poultry health, namely ORT and PM. The rationale behind selecting these pathogens was the fact that ORT has relatively recently been recognized as an increasing problem in the industry but little of its epidemiology is known, while PM has been more extensively studied with its epidemiology being well described in comparison. Our results showed that, considering a similar time frame and using data from a single production company, a higher number of genetic clusters was found for PM compared to ORT. This finding is in concordance with a recent study that characterized multiple PM strains from isolates from a single outbreak for one commercial turkey company [2]. In addition, our findings are supported by the recent work on the pathogenic adaptation and genetic divergence of PM, showing high strain diversity and plasticity [41]. This is typical for a primary pathogen which can be introduced into and spread in a population within a relatively short time and limited geographical area.

Our second main finding was that spatio-temporal clusters were found for PM, but not for ORT. This was unsurprising, since there are early reports on the existence of spatial-temporal clusters of fowl cholera in the literature [31,41]. The lack of clear spatio-temporal clusters for ORT is also consistent with its suggested main role as an opportunistic secondary pathogen [42] but studies investigating spatio-temporal clustering for ORT are currently limited. The absence of spatial-temporal clusters for ORT in this study may be explained by the lower genetic diversity observed for ORT. This finding is in line with a previous study that concluded that the population structure of 55 ORT isolates was primarily clonal, with repeated recovery of isolates with identical genotypes even when considering large geographical areas and long time periods [43]. Moreover, recent studies have reported low genetic diversity among ORT isolates [20,44]. This is in clear contrast to our results on PM, which resulted in 13 genetic clusters from only 69 isolates over 9 years compared to the 3 clusters found for ORT. It has been suggested that ORT is likely to have been recently introduced and dispersed into poultry populations worldwide [44]. This rapid spread of ORT may be locally and regionally facilitated through the movements of eggs [45], farm-to-farm spread via aerosols, and direct contact with wild fowl and water [25,44], leaving relatively homogenous and stable ORT populations across larger geographic regions. Indeed, the lack of a strong correlation between allelic differences and the geographic distance between outbreak location for ORT in our study adds to the suggestion that regional ORT strains are frequently transmitted between commercial turkey sites while maintaining a low genetic diversity.

This study does not come without limitations. First, because we relied on retrospectively obtained isolates, we did not have access to further site-level or flock-level information; for example, those related to biosecurity and management, which prevented further risk analysis and contextualization of isolates. In addition, the space–time permutation used does not account for the population at risk as it potentially changes over the investigated time period of the study. It is also likely that not all suspected outbreaks were accurately reported as ORT outbreaks as the disease severity and signs may vary significantly between flocks [22,25]. However, this was not the main objective of this project. Additionally, the dataset contained isolates from a single production company; therefore, it was not comprehensive of all potential PM and ORT clinical manifestations in the area. However, it is estimated that the vast majority of commercial turkey sites in the region were represented within our participant. Furthermore, it is important to note that the isolates were obtained from a veterinary diagnostic laboratory bank and, considering the potential challenges related to the culture and isolation of bacteria, there could have been isolates that were missed over the years due to issues with diagnostic detections and storage. This is a common issue with retrospective studies and a challenge difficult to overcome when dealing with long periods of time; therefore, the results should be interpreted with caution. It is also important to note that all the isolates were maintained in a single veterinary diagnostic laboratory and, even though this contributed to the consistency of standard operating procedures for our working dataset, it does limit the generalization of the study results since other geographical regions were not represented herein.

## 5. Conclusions

This study revealed the regional spatio-temporal clusters for PM in commercial turkey sites between 2013 and 2021. The correlations between allele diversity and distances between the outbreak locations were weak and did suggest continuous in-between site transfers for ORT but not for PM. However, the presence of genetic clusters in a limited geographical area is suggestive of a primary pathogen route for PM using localized farm-to-farm spread.

## Data Availability

De-identified data and non-proprietary data may be accessed by request.

## Supplementary Materials

The following supporting information can be downloaded at: https://www.mdpi.com/article/10.3390/pathogens12081004/s1, Supplementary Table S1. Median allelic differences for Ornithobacterium rhinotrachealis (ORT) and Pasteurella multocida (PM) isolates between sampling year and within clusters derived from a minimum spanning tree analysis. Ni and Nc represent the number of available isolates and the number of 1:1 allelic comparisons between isolates for each year and cluster, respectively. The allelic difference between isolates is provided as median, min and max. A high median value represents a large allelic diversity between isolates within year or cluster; Supplementary Table S2. Median allelic differences for Pasteurella multocida (PM) isolates between sampling year and within clusters derived from a minimum spanning tree analysis. Ni and Nc represent the number of available isolates and the number of 1:1 allelic comparisons between isolates for each year and cluster, respectively. The allelic difference between isolates is provided as median, min and max. A high median value represents a large allelic diversity between isolates within year or cluster; Supplementary Table S3. Ornithobacterium rhinotrachealis (ORT) and Pasteurella multocida (PM) genomes sourced from GenBank and from collected Turkey isolates used for the development of the cgMLST schemes and further isolate characterization in the present study.
