# Supplementary material for: Space–Time Patterns of Poultry Pathogens in the USA: A Case Study of Ornithobacterium rhinotracheale and Pasteurella multocida in Turkey Populations
Source: Pathogens. 2023 Jul 31;12(8):1004. doi: 10.3390/pathogens12081004 (PMC10460037; doi:10.3390/pathogens12081004)
Supplement: Supplementary file 1 [file pathogens-12-01004-s001.zip › pathogens-2455092-SI.pdf]

Supplementary Table S1. Median allelic differences for *Ornithobacterium rhinotrachealis* (ORT) and *Pasteurella multocida* (PM) isolates between sampling year and within clusters derived from a minimum spanning tree analysis. Ni and Nc represent the number of available isolates and the number of 1:1 allelic comparisons between isolates for each year and cluster, respectively. The allelic difference between isolates is provided as median, min and max. A high median value represents a large allelic diversity between isolates within year or cluster.

ORT

Year	N_i	N_c	Median	Min	Max
**2013**	1	n/a	n/a	n/a	n/a
**2014**	3	6	19	0	36
**2016**	3	6	1	1	2
**2018**	29	812	484	0	517
**2019**	18	306	50	0	512
**2020**	14	182	15	0	512
**2021**	31	930	20	0	703

Cluster	N_i	N_c	Median	Min	Max
**1**	4	12	19	0	36
**2a**	28	756	8	0	69
**2b**	44	1892	10	0	72
**3**	23	506	2	0	10

Supplementary Table S2. Median allelic differences for *Pasteurella multocida* (PM) isolates between sampling year and within clusters derived from a minimum spanning tree analysis. Ni and Nc represent the number of available isolates and the number of 1:1 allelic comparisons between isolates for each year and cluster, respectively. The allelic difference between isolates is provided as median, min and max. A high median value represents a large allelic diversity between isolates within year or cluster.

PM

Year	N_i	N_c	Median	Min	Max
**2013**	1	n/a	n/a	n/a	n/a
**2014**	3	6	1232	23	1233
**2015**	4	12	1217	0	1234
**2016**	11	110	1163	19	1235
**2017**	26	650	1098	13	1182

**2018**	14	182	1095	3	1183
**2019**	3	6	6	3	7
**2020**	2	2	1117	1117	1117
**2021**	4	12	1212	143	1227

**Cluster**	**N_i**	**N_c**	**Median**	**Min**	**Max**
**1**	16	240	26	3	52
**2**	12	132	106	13	144
**3**	7	42	63	3	96
**4**	6	30	30	11	62
**5**	5	20	72	0	143
**6**	4	12	98	14	115
**7**	3	6	40	25	44
**8**	3	6	35	34	37
**9**	3	6	27	9	30
**10**	2	1	40	40	40
**11**	2	1	58	58	58
**12**	2	1	33	33	33
**13**	2	1	13	13	13

Supplementary Table S3. *Ornithobacterium rhinotrachealis* (ORT) and *Pasteurella multocida* (PM) genomes sourced from GenBank and from collected Turkey isolates used for the development of the cgMLST schemes and further isolate characterization in the present study.

Pathogen	Isolate ID	SRA/Nucleotide Accessions number	Sequence Source
Ornithobacterium	Sample_57	SRR25316877	This Study
	14-5910	SRR25316876	This Study
	Sample_81	SRR25316870	This Study
	10746-14	SRR25316872	This Study
	240_OR7	SRR25316873	This Study
	246_OR13	SRR25316874	This Study
	247_OR_14	SRR25316857	This Study
	249_OR_16	SRR25316858	This Study
	25._Sample_122	SRR25316859	This Study
	255_OR_22	SRR25316860	This Study

	263_OR30	SRR25316875	This Study
	41._Sample_161	SRR25316861	This Study
	53._Sample_191	SRR25316862	This Study
	61._Sample_210	SRR25316863	This Study
	G203_S37	SRR25316864	This Study
	ORT_109_S15	SRR25316865	This Study
	ORT_64_S20	SRR25316866	This Study
	Shen_1_sample_97	SRR25316867	This Study
	UC_17_S29	SRR25316868	This Study
	UC_18_S40	SRR25316869	This Study
	UC_2_S72	SRR25316871	This Study
	41._ORT41	SRR8654981	Downloaded from GenBank
	8._ORT8	SRR8654988	Downloaded from GenBank
	DSM 15997	SRR3926759	Downloaded from GenBank
	ORT105	SRR10600831	Downloaded from GenBank
	ORT109	SRR10600826	Downloaded from GenBank
	ORT113	SRR10600822	Downloaded from GenBank
	ORT116	SRR10600819	Downloaded from GenBank
	ORT118	SRR10600817	Downloaded from GenBank
	ORT126	SRR10600808	Downloaded from GenBank
	ORT133	SRR10600800	Downloaded from GenBank
	ORT149	SRR10600782	Downloaded from GenBank
Pasteurella multocida	6717	SRR25320037	This Study
	6814	SRR25320038	This Study
	7602	SRR25320049	This Study
	9170	SRR25320057	This Study
	9186	SRR25320036	This Study
	9320	SRR25320035	This Study
	9334	SRR25320034	This Study
	9455	SRR25320030	This Study
	9710	SRR25320029	This Study
	10081	SRR25320028	This Study
	10166	SRR25320039	This Study
	10253	SRR25320040	This Study
	10342	SRR25320041	This Study
	10378	SRR25320042	This Study
	10516	SRR25320043	This Study
	10757	SRR25320044	This Study
	10848	SRR25320045	This Study
	10976	SRR25320046	This Study
	11006	SRR25320047	This Study
	11145	SRR25320048	This Study
	11244	SRR25320050	This Study
	28921	SRR25320051	This Study
	28936	SRR25320052	This Study
	14-1155-2	SRR25320053	This Study

	15-15053	SRR25320054	This Study
	16-18953	SRR25320055	This Study
	17-17161-1	SRR25320031	This Study
	18-18824-1	SRR25320032	This Study
	CU-1	SRR25320033	This Study
	SEPTICA	SRR25320056	This Study
	VAC1	SRR25320059	This Study
	VAC9	SRR25320058	This Study
	PM1614	SRR10485187	Downloaded from GenBank
	5	SRR5192694	Downloaded from GenBank
	10027	SRR5192725	Downloaded from GenBank
	9486	SRR5192656	Downloaded from GenBank
	9719	SRR5192632	Downloaded from GenBank
	6054	SRR5192685	Downloaded from GenBank
	6630	SRR5192679	Downloaded from GenBank
	9832	SRR5192621	Downloaded from GenBank
	10045	SRR5192722	Downloaded from GenBank
	9673	SRR5192640	Downloaded from GenBank
	9821	SRR5192623	Downloaded from GenBank
	Liver3	SRR5192588	Downloaded from GenBank
	9613	SRR5192645	Downloaded from GenBank
	9237	SRR5192664	Downloaded from GenBank
	9904	SRR5192608	Downloaded from GenBank
	9955	SRR5192602	Downloaded from GenBank
	9488	SRR5192655	Downloaded from GenBank
	10086	SRR5192712	Downloaded from GenBank
	9612	SRR5192646	Downloaded from GenBank
	9621	SRR5192644	Downloaded from GenBank
	9981	SRR5192594	Downloaded from GenBank
	P-1591	SRR5192581	Downloaded from GenBank
	6114	SRR5192681	Downloaded from GenBank
	9847	SRR5192617	Downloaded from GenBank
	9605	SRR5192647	Downloaded from GenBank
	9185	SRR5192668	Downloaded from GenBank
	5864	SRR5192689	Downloaded from GenBank
	9224	SRR5192665	Downloaded from GenBank
	9846	SRR5192618	Downloaded from GenBank
	9242	SRR5192663	Downloaded from GenBank
	9972	SRR5192598	Downloaded from GenBank
	10088	SRR5192711	Downloaded from GenBank
	9464	SRR5192657	Downloaded from GenBank
	9956	SRR5192601	Downloaded from GenBank
	10067	SRR5192714	Downloaded from GenBank
	10046	SRR5192721	Downloaded from GenBank
	9866	SRR5192616	Downloaded from GenBank
	10042	SRR5192723	Downloaded from GenBank

	P-1662	SRR5192580	Downloaded from GenBank
	9599	SRR5192649	Downloaded from GenBank
	9076	SRR5192671	Downloaded from GenBank
	6846	SRR5192675	Downloaded from GenBank
	10091	SRR5192710	Downloaded from GenBank
	9102	SRR5192669	Downloaded from GenBank
	10049	SRR5192720	Downloaded from GenBank
	9198	SRR5192666	Downloaded from GenBank
	P-1059-1	SRR5192570	Downloaded from GenBank
	P-1702	SRR5192579	Downloaded from GenBank
	P-2192	SRR5192575	Downloaded from GenBank
	P-1997	SRR5192578	Downloaded from GenBank
	P-1581	SRR5192582	Downloaded from GenBank
	P-2095	SRR5192577	Downloaded from GenBank
	P-2100	SRR5192576	Downloaded from GenBank
	P-903	SRR5192571	Downloaded from GenBank
	P-1573	SRR5192583	Downloaded from GenBank
	P-2225	SRR5192574	Downloaded from GenBank
	P-2237	SRR5192573	Downloaded from GenBank
	P-2723	SRR5192572	Downloaded from GenBank
	M-1404	SRR5192586	Downloaded from GenBank
	P1059-gallicida	CM001581.1	Downloaded from GenBank
	FDAARGOS_218	NZ_CP020405.2	Downloaded from GenBank
	RCAD0259	CP014157.1	Downloaded from GenBank
	Ban-PM4	CP052764.1	Downloaded from GenBank
	X-73	NZ_CM001580.1	Downloaded from GenBank
